# Epidemiology, Genotype Distribution, Prognosis, Control, and Management of Viral Hepatitis B, C, D, and Hepatocellular Carcinoma in Mongolia

**DOI:** 10.5005/jp-journals-10018-1260

**Published:** 2018-05-01

**Authors:** Oidov Baatarkhuu, Tsagaantsooj Gerelchimeg, Dashchirev Munkh-Orshikh, Badamnachin Batsukh, Ganbold Sarangua, Jazag Amarsanaa

**Affiliations:** 1 Department of Infectious Diseases, Mongolian National University of Medical Sciences, Ulaanbaatar, Mongolia; Mongolian Association for the Study of Liver Diseases, Ulaanbaatar, Mongolia; 2 Department of Infectious Diseases, Mongolian National University of Medical Sciences, Ulaanbaatar, Mongolia; National Center for Communicable Diseases, Ulaanbaatar, Mongolia; 3 Mongolian Association for the Study of Liver Diseases, Ulaanbaatar, Mongolia; Happy Veritas Clinic and Diagnostic Center, Ulaanbaatar, Mongolia; 4 Department of Internal Medicine, Yonsei University College of Medicine, Seoul, South Korea

**Keywords:** Genotype, Hepatitis B virus, Hepatitis C virus, Hepatitis D virus, Hepatocellular carcinoma, Mongolia, National program.

## Abstract

Mongolia is located between Russia and China. The total population of Mongolia as of December 2017 is estimated to be 3.2 million people. According to our previous study results, the prevalence of HBV was 11.8%, and anti-HDV was detected in 4.8% among the HBsAg-positive subjects. Interestingly, most HCV infection is caused by genotype 1b. Among all HBV DNA-positive samples, 98.5% were classified into genotype D, and regarding HDV genotypes, all HDV RNA-positive samples, 100%, were classified into genotype I.

The second study is the baseline survey of a Nationwide Cancer Cohort Study. Prevalence of HBsAg was 10.6%. Additionally, HCV infection was observed in 9.9%, and 0.8% were coinfected with HBV and HCV among the general population aged from 10 to 64 years.

The third study investigated the population-based prevalence of hepatitis B and C virus in apparently healthy population of Ulaanbaatar city, Mongolia. The anti-HCV prevalence was 9.0%. In addition, the prevalence of HBV was 8.0%.

The fourth study is on the prevalence of HCV and coinfections among nurses in a tertiary hospital in Mongolia. The prevalence of HCV was 18.9%. Additionally, HBV infection was observed in 23.1%, and 1.2% were coinfected with HCV and HBV.

Mongolia has the highest HCC incidence in the world (78.1/100,000, 3.5* higher than China).

As a result, the Mongolia government has launched The National Viral Hepatitis Program, which is a comprehensive program that involves all aspects from prevention to care and disease control to meet a reduction goal for morbidity and mortality due to HBV, HCV, and HDV. Consequently, access to antiviral therapies is now improving in Mongolia.

**How to cite this article:** Baatarkhuu O, Gerelchimeg T, Munkh-Orshikh D, Batsukh B, Sarangua G, Amarsanaa J. Epidemiology, Genotype Distribution, Prognosis, Control, and Management of Viral Hepatitis B, C, D, and Hepatocellular Carcinoma in Mongolia. Euroasian J Hepato-Gastroenterol 2018;8(1):57-62.

## INTRODUCTION

In 2017, during the European Association for the Study of the Liver congress, the World Health Organization (WHO) reported on the updated global prevalence of HCV and HBV infections. In 2015, 71 million persons worldwide were living with HCV infection. Overall, in 2015, the global prevalence of HCV infection was 1%. Genotypes 1 and 3 were the most common causes of HCV infection worldwide.

In 2015, the global prevalence of HBV infection in the general population was 3.5%. Prevalence was the highest in the African countries at 6.1% and Western Pacific Regions at 6.2%. Overall, approximately 257 million persons worldwide are living with HBV infection.^1^

Mongolia has the highest prevalence of HBV, HCV, and HDV infection. Mongolia had introduced HBV vaccination into routine immunization program for newborns and children under 1 year of age since 1991, which substantially decreased the incidence of HBV infection.^2^ In addition, a randomized controlled trial showed that the drug tenofovir disoproxil fumarate can safely prevent vertical transmission of HBV, and could further improve this decrease in HBV infection.^3^ The WHO supported a series of national serological surveys and found that Mongolia had reached its regional goal, with 82% of children fully vaccinated as of 2010.^4^ The HBsAg carrier rate was 0.53% among children aged 4 to 6 years in 2009 to 2010 in Mongolia.^4^ However, the prevalence of HBV is still high among adults in Mongolia. A review of studies from 2000 to 2011 found an HBV seroprevalence of 11.8% in the unvaccinated population.^2^ Approximately 13.6% of those who are HBsAg positive also have coinfection with HDV, which speeds up the progression of liver disease.^5-7^

The prevalence of HCV was 15.6%.^8^ Although HCV can now be cured with new direct-acting antiviral (DAA) therapies, the morbidity and mortality are still high, due to delayed diagnosis and poor access to newer medications. The seroprevalence of dual infection with HCV and HBV was found to be in the range from 5.3 to 22.9% in the published literature.^8-14^

Mongolia has the highest HCC incidence in the world (78.1/100,000, 3.5x higher than China).^2^ The number one cause of cancer-related death was HCC, responsible for 44% of male and 42% of female cancer deaths.^8,15^

In addition, data from the National Cancer Registry of Mongolia found that the leading cause of cancer was also HCC in both genders (41% of male cancers, 33% of female cancers).^15^ In 2014, the liver cancer morbidity and mortality were 64.4 and 47.4 per 100,000 persons respectively, according to the Center for Health Development’s Health Indicators report.^16,17^

As a result, the Mongolian government has launched The National Viral Hepatitis Program, which is a comprehensive program that involves all aspects from prevention to care and disease control to meet a reduction goal for morbidity and mortality due to HBV, HCV, and HDV. Consequently, access to antiviral therapies is now improving in Mongolia.^4^

## PREVALENCE AND GENOTYPE DISTRIBUTION, PROGNOSIS OF HEPATITIS B, C, AND D VIRUS INFECTIONS

Mongolia is a unique country with high endemicity for three blood-borne hepatitis viruses, namely HBV, HCV, and HDV. Viral hepatitis B and C are one of the major causes of liver cirrhosis and HCC in Mongolia. We have completed the study named “Prevalence and genotype distribution of hepatitis B and C virus among apparently healthy populations in Mongolia: a population-based nationwide study.” This study population consisted of 1,512 subjects from 13 provinces and Ulaanbaatar city, which is the capital city of Mongolia. The age ranged from 0 to 80 years. According to our study results, the prevalence of anti-HCV was 15.6%, and the HCV RNA was detected in 11.0% among the anti-HCV-positive cases.^4^ In addition, the prevalence of HBV was 11.8%, and anti-HDV was detected in 4.8% among the HBsAg-positive subjects. The prevalence of anti-HCV and HCV RNA had a tendency to increase with age. The prevalence of anti-HCV and HCV RNA in population aged over 61 years was significantly higher than those aged 31 to 40 years. The history of dental care, surgery, and tattooing was significantly more frequent in anti-HCV and HBsAg-positive subjects compared with anti-HCV and HBsAg-negative subjects in this study. The history of blood transfusion, dental care, surgery, and tattooing was significantly more frequent in anti-HDV-positive subjects compared with anti-HDV-negative subjects.

Interestingly, most HCV infection is caused by genotype 1b. On the contrary, genotype 2 of HCV is very rare, less than 2% in Mongolia. Among all HBV DNA-positive samples, 98.5% were classified into genotype D, and the remaining 1.5% were genotype C. Regarding HDV genotypes, all HDV RNA-positive samples, i.e., 100%, were classified into genotype I. The extreme predominance of HCV genotype 1b in the Mongolian population may be explained by the greater ethnic and genetic homogeneity of the current Mongolian population.

The second study is the baseline survey of a Nationwide Cancer Cohort Study. A population-based national cross-sectional survey was conducted with multistage random cluster sampling. Population aged from 10 to 64 years in UB city and four geographic regions were randomly selected. Among study subjects, 37.2% were from rural provinces and 39.2% were men. Prevalence of HBsAg was 10.6%; additionally, HCV infection was observed in 9.9, and 0.8% were coinfected with HBV and HCV among general population aged 10 to 64 years.^18^ The prevalence of HCV and HBV infection decreased from 15.6 to 9.9% and 11.8 to 10.6% respectively, among the apparently healthy individuals (AHI) in Mongolia during the last decade.

The third study investigated the population-based prevalence of HBV and HCV in an apparently healthy population of Ulaanbaatar city, Mongolia.^19^ Totally, 2,667 people who live in Ulaanbaatar were included in this study. The rapid immunochromatographic test was applied for detection of HCV antibodies and HBV rapid test. The mean age of the subjects was 38 + 12 years, and 1,064 (51%) were male. The anti-HCV prevalence was 9.0%. In addition, the prevalence of HBV was 8.0%. The mean age of HBV infection was 39 + 12.0 years.

Overall, according to the 2008 study conducted by Dr Oidov Baatarkhuu, the prevalence of anti-HCV in Ulaanbaatar was 13.7% and HBV was 9.3%. Between 2015 and 2016, studies conducted by Dr Dashmaa Tungalag, Oidov Baatarkhuu, and Dr Jazag Amarsanaa showed that the prevalence of anti-HCV decreased from 13.7 to 9.0% and HBV decreased from 9.3 to 8.0% in apparently healthy populations in Ulaanbaatar, Mongolia.

The fourth study is prevalence of HCV and coinfections among nurses in a tertiary hospital in Mongolia.^20^ This study was conducted in a two-staged random cluster sampling. The study population consisted of 598 nurses from five hospitals. The prevalence of HCV was 18.9%. Additionally, HBV infection was observed in 23.1%, and 1.2% were coinfected with HCV and HBV.

Hepatitis D infection is highly endemic among subjects with chronic HBV infection in Mongolia. The likely reason is the association between high HBV prevalence, the existence of HDV in the community, and blood-borne virus transmission in health and nonhealth settings over the past decades. The prevalence of anti-HDV among HBsAg-positive individuals indicates a likely HBV/ HDV coinfection ranging from 41 to 67% in the two major seroepidemiological studies.^21,22^ The HBV/HDV coinfection is associated with more rapid progression of liver disease than is seen in HBV monoinfection. For example, a 2005 study of patients with chronic liver disease reported a prevalence of HDV RNA of 39% among patients with chronic hepatitis, 51% among those with liver cirrhosis, and 80% among those with HCC.

## NATIONAL CANCER REGISTRY, EPIDEMIOLOGY, PREVENTION, MANAGEMENT, AND CONTROL FOR EARLY DETECTION OF HCC IN MONGOLIA

The National Cancer Registry was established in 1971. Its data sources include 21 provincial hospitals, 9 district hospitals, 16 other specialized hospitals and centers, the National Pathology Center, the Breast and Cervical Cancer Screening Registry, and hospices. Quarterly information on cancer cases is reported to the National Cancer Center on form AM57. There is a cancer registrar in each district and province who collates this information. Reporting is a combination of manual and electronic (Excel). Line-by-line data are available and aggregation is central, so analysis (e.g., demographics, diagnosis, histological confirmation, etc.) is possible.

Since 2007, the National Cancer Registry office has tried to work with the Mongolian Civil Registry Office on linkage with mortality data. At the national level, the registry has had difficulty in linking cancer incidence data with death information due to lack of access to data. The Registry is now working with the provincial civil registries to match cancer with mortality data on an annual basis.

The Registry has already translated the International Classification of Disease 10 manual into Cyrillic. They would like to translate the manual for tumor-node-metastasis classification of malignant tumors, and have asked for help in contacting the Union for International Cancer Control for permission to translate.

Cancer deaths are reported to the Cancer Registry, as well as the Civil Registry. Back-investigation also occurs to investigate and confirm cancer as a cause of death.

The Strategy for Early Detection of Liver Cancer issued in May 2014 (commenced in 2015) recommends screening people 40 to 65 years of age for HBV and HCV with rapid tests. Confirmation by enzyme-linked immunosorbent assay will be done at the district level. Those identified with chronic hepatitis will receive alfa-fetoprotein (AFP) and ultrasound tests every 6 months. Data will be collated at the National Cancer Center. Those identified with tumors smaller than 2 cm will be followed up every 4 months, which differs from American and European guidelines.^23,24^

Patients with lesions larger than 2 cm will be sent to the National Cancer Center. There is an algorithm to indicate the need for biopsy (e.g., AFP level, ultrasound findings).

**Graph 1:**
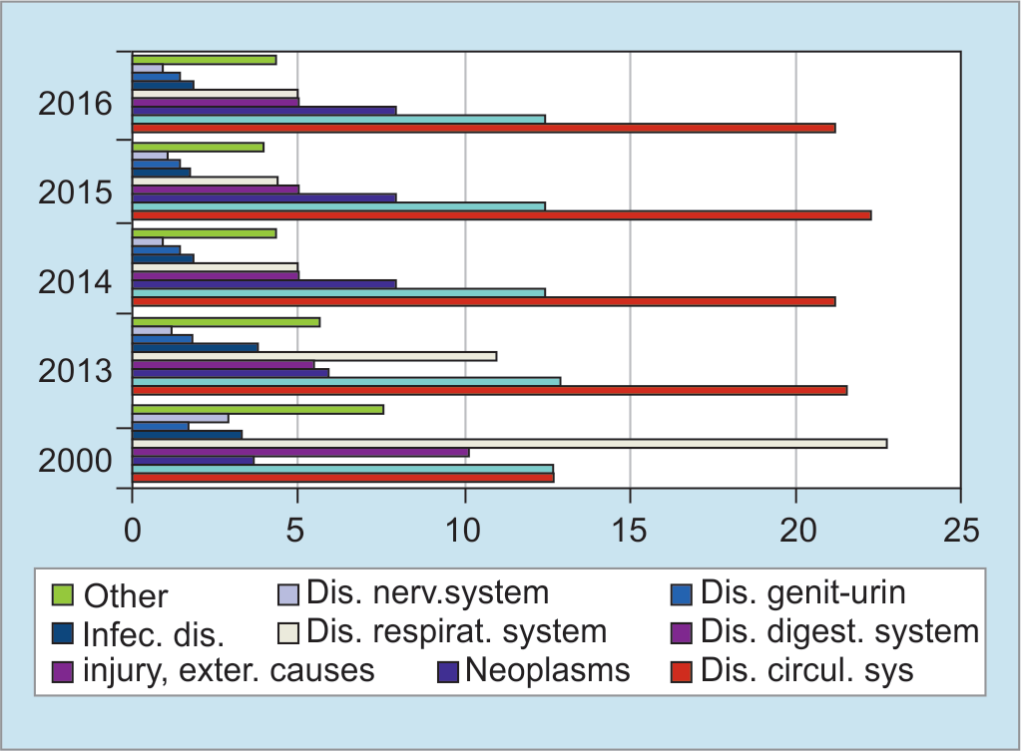
Common causes of mortality 2000-2016

92 to 95% of HCC patients in Mongolia are related with HBV and HCV infections occurring in 115 cases per 100,000 people per year. Mongolia, where approximately 3.2 million people live, has around 2000 to 2200 new cases of HCC every year. Graph 1 shows the common causes of mortality in Mongolia. In the last 16 years, cancers have always been in the second place among causes of mortality in Mongolia (Graph 1).

**Graph 2:**
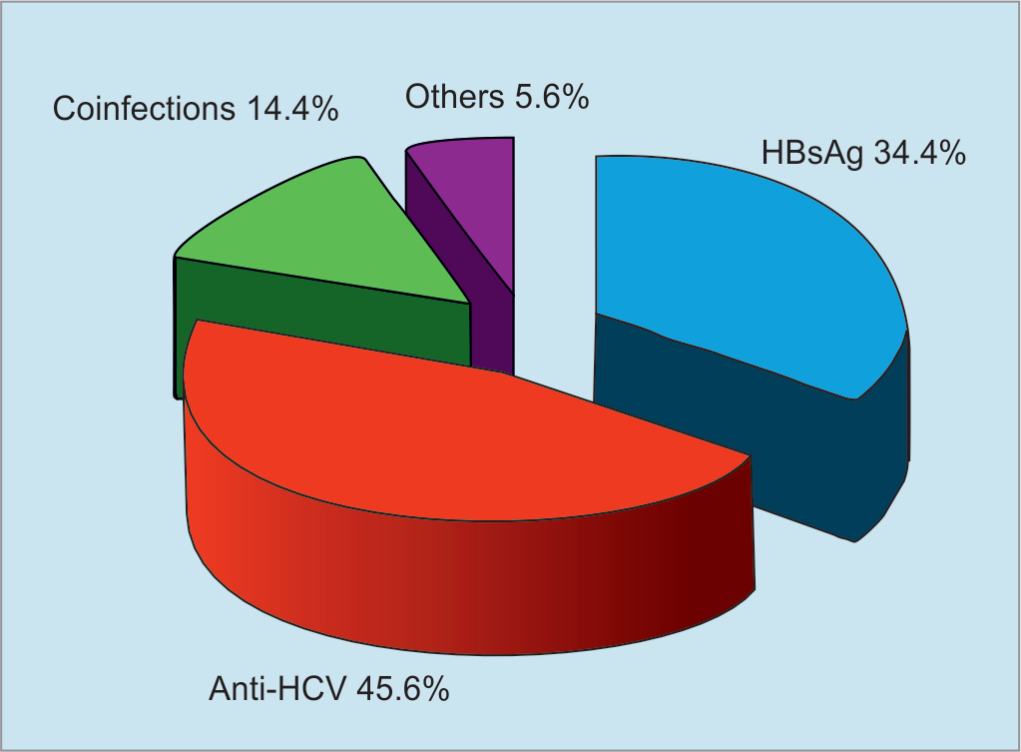
Prevalence of hepatitis virus markers in patients with HCC

According to the 2016 national statistics of Mongolia, liver cancer is the most prevalent malignancy, which is equal to 44.0% of all cancers in Mongolia.

The study population consisted of 195 subjects from four hospitals in Ulaanbaatar city of Mongolia, and the age ranged from 30 to 86 years. Most of the patients were Khalkh, which is the main ethnic group in Mongolia.^25^

The most common etiology for HCC in our patients was HCV infection, which is 46%, HBV infection 34%, coinfection of HBV and HCV 14%, and alcohol 5.6% (Graph 2). The risk factors associated with HCC development were history of acute hepatitis, chronic hepatitis, and the presence of liver cirrhosis. Of these, the presence of liver cirrhosis was the strongest risk factor.

In Mongolia, over 60% of patients had a tumor size more than 5 cm. Single tumors were only found in 15%. The mean AFP level was 196 ng/mL. In 18.5%, distant metastasis existed. Regarding tumor stage, there was no patient with stage 1 disease. In addition, most patients with HCC were diagnosed in the advanced stage.

In Mongolia, the treatment modality is very limited. According to the results of our study, 14% of patients received surgical resection and their survival was the best. About 11.8% of patients received radiofrequency ablation and their survival was 11 months. About 55% of patients received transarterial chemoembolization (TACE) and their median survival was 17 months. The prognosis for patients with supportive care was very poor, with a median survival of 5 months. About 20% of patients received only supportive care because of advanced disease.

Regarding cause of death, about 50% patients died of HCC progression and the others died of liver failure or gastrointestinal bleeding.

Regarding the current capacity for early detection of HCC in Mongolia, AFP is available in all hospitals except inter-soum and soum’s hospitals. Ultrasound is available in all areas. Computed tomography, angiography, and TACE are very limitedly available; they can be used at national cancer centers, state and central hospitals, and some private clinics in Ulaanbaatar city (Table 1).

## NATIONAL STRATEGY ON VIRAL HEPATITIS CONTROL 2010 TO 2015

Mongolia has a large burden of viral hepatitis, especially chronic HBV and HCV infections, which are associated with cancer and cirrhosis. Chronic HBV infection is acquired in early childhood in Mongolia, while HCV and HDV transmissions are healthcare related. Mongolia also has the highest, and increasing, rate of liver cancer and mortality from liver cancer in the world. The HCC is the second most common cause of death in Mongolia, and liver cancer is responsible for 44% of all cancers. Chronic hepatitis B and C infections are responsible for 95% of liver cancers in the country.

There have been successes in responding to the epidemic. Mongolia’s National Strategy on Viral Hepatitis Control covered the period from 2010 to 2015 and proposed five objectives, with the overarching aim of reducing the incidence of viral hepatitis to 10 cases per 10,000 by 2015. This goal has now been achieved. The prevalence of HB-sAg among 4- to 6-year-olds has now met the regional goal of <1.0%. In addition, the introduction of vaccination against the hepatitis A virus (HAV) into routine vaccination schedules has had a substantial impact on reducing the proportion of acute jaundice cases related to HAV infection. The HCV and HDV transmission appears to have decreased substantially over the past 20 years as infection control systems have improved.

**Table 1:** **Table 1:** Current capacity for early detection of HCC

*Early detection tests*		*NCC and hospital 1,2,3*		*Regional diagnostic and treatment centers*		*Aimag and district hospitals*		*Inter-soum and soum hospitals*	
Alpha-fetoprotein testing		+		+		+		–	
Ultrasound		+		+		+		+	
Liver cancer Computed tomography		+		+		–		–	
Angiography		+		+		–		–	
Cytology		+		+		+		–	

Given the disease burden, and also the progress made in responding to viral hepatitis, a review was undertaken to assess the current situation and response in Mongolia. The review team visited public and private facilities providing prevention, surveillance, diagnosis, care, and treatment services for hepatitis and liver cancer in Ulaanbaatar and Bulgan province. Infection control practices were also reviewed at these services. In addition, a meeting with key stakeholders was held on September 12, 2014, and a consensus meeting to discuss recommendations from the initial review was held on January 21 and 22, 2015. The consensus meeting provided an opportunity to hold a workshop on the content of the new national hepatitis strategy, later to be named the Viral Hepatitis Prevention, Control, and Elimination Program.

With new developments in hepatitis C care and treatment, the Ministry of Health and Sports initiated policy discussions with the Parliamentary Standing Committee on Social Policy, Education, Culture and Sciences. The WHO provided technical support during this policy dialogue and proposed to have a multistakeholder dialogue to discuss viral hepatitis financing options in Mongolia. The financing dialogue concluded the necessity of conducting economic analysis of hepatitis B and C care and treatment. The WHO in collaboration with the Ministry of Health and Sports and the Centre for Disease Analysis completed hepatitis C burden and economic modeling exercises.

## HEPATITIS PREVENTION CONTROL AND ELIMINATION PROGRAM, 2016 TO 2020

Recently, the Parliament of Mongolia officially approved the implementation of the Hepatitis Prevention Control and Elimination Program (HPCEP) between 2016 and 2020. The current mission of the HPCEP is to eliminate HCV in Mongolia by 2020. Also, the aim is to significantly decrease the incidence of viral hepatitis, liver cirrhosis, and HCC. The government of Mongolia allocated 232 billion Mongolian tugrug or 96 million USD for the HPCEP through 2020.

The first stage of the program is screening the high-and moderate-risk population for HCV and HBV as follows:

- To screen hepatitis B and C among general populations from 40 to 65 years (2017).
- To screen hepatitis B and C among general populations from 15 to 40 years (2018).

Treatment campaign started from January 2016. At present, 18,000 patients with HCV had been treated with the brand Harvoni and generics. Since 2016, there were four generic companies and brand Gilead Sciences Inc. manufacturing HCV DAAs in Mongolia and branded Sofosbuvir/Ledipasvir (SOF/LDV) is priced $300 per month and generic SOF/LDV price is $150 per month.

By the end of 2016, the Mongolian government had included HBV and HCV medicines in the national health insurance. The health insurance will provide $75 for one bottle of both generic and brand SOF/LDV.

As of 2017, in the first 6 months, approximately 18,000 people have been treated by the new DAAs. The cure rate for treatment of HCV infection was 96 to 99%.^24^

## CONCLUSION

- The prevalence of HCV and HBV infection has decreased from 15.6 to 9.9% and 11.8 to 10.6% respectively, among the AHI in Mongolia during the last decade.
- The prevalence of HCV and HBV infection has decreased from 13.7 to 8.0% and 9.3 to 9.0% among the AHI in Ulaanbaatar.
- Thus, there is the need for efforts to improve infection control measures in the medical system in Mongolia.
- The past infection of viral hepatitis B and C is the main cause of HCC in Mongolia. The most common etiology for HCC in Mongolia was HCV infection, which is 46%, HBV infection 34%, coinfection of HBV and HCV 14%, and alcohol 5.6%.
- The prognosis of HCC was poor, so more than 50% died within the second year after the diagnosis of HCC in Mongolia.
- The current mission of the HPCEP is to eliminate HCV in Mongolia by 2020 and significantly decrease the incidence of viral hepatitis, liver cirrhosis, and HCC.
